# Clinical impact of microbleeds in patients with Alzheimer’s disease

**DOI:** 10.1186/s12877-022-03456-y

**Published:** 2022-09-29

**Authors:** Daniel Vázquez-Justes, Iván Aguirregoicoa, Leandre Fernandez, Anna Carnes-Vendrell, Faride Dakterzada, Laura Sanjuan, Andreu Mena, Gerard Piñol-Ripoll

**Affiliations:** 1 Neurology Department, Clinical Neuroscience Research Group, IRBLleida-Hospital Universitari Arnau de Vilanova, Lleida, Spain; 2 Radiology Department, Hospital General Barbastro, Salud Aragón, Barbastro, Spain; 3 Radiology Department, Hospital Universitari Santa Maria, IRBLleida, Lleida, Spain; 4 Unitat Trastorns Cognitius, Clinical Neuroscience Research, Hospital Universitari Santa Maria, IRBLleida, Lleida, Spain; 5 Cognitive Disorders Unit, Hospital Universitari Santa Maria, Rovira Roure n° 44. 25198, Lleida, Spain

**Keywords:** Cognitive decline, Microbleeds, Stroke, Death, Alzheimer’s disease

## Abstract

**Introduction:**

Cerebral microbleeds (CMBs) are more frequent in patients with Alzheimer’s disease (AD) than in the general population. However, their clinical significance remains poorly understood. We carried out a multimodal approach to evaluate the impact of CMBs at a clinical, neuropsychological, and survival level, as well as on core AD biomarkers in the cerebrospinal fluid (CSF) in AD patients.

**Methods:**

We prospectively recruited 98 patients with mild-moderate AD. At baseline, they underwent brain MRI, and AD CSF biomarkers and APOE genotypes were analysed. An extensive neuropsychological battery was performed at baseline and after 1 year of follow-up. We analysed the stroke incidence and mortality with survival analyses.

**Results:**

Forty-eight (48.5%) patients had at least one CMBs. Eight (8.2%) patients had strictly nonlobar CMBs, 39 (40.2%) had any lobar CMB locations. The incidence of stroke was higher in AD patients with lobar CMBs than in those without CMBs (*p* < 0.05). Mortality did not differ among groups (*p* > 0.05). At the cognitive level, CMBs patients deteriorated more rapidly at 12 months according to MMSE scores, with no differences observed at 24 months. We did not observe differences in the other tests, except for an increase in caregiver burden in the CMBs group. The presence of cerebral amyloidosis and APOE **ε**4 were associated with a greater presence of CMBs.

**Conclusion:**

CMBs are associated with an increased risk of ischemic stroke in AD patients without differences in mortality. Patients with CMBs did not seem to have different consequences associated with cognitive decline except for an increase in caregiver overload.

## Introduction

Alzheimer’s disease (AD) is the most frequent type of dementia, and the number of affected patients is expected to increase in the coming years [1]. The neuropathologic hallmarks include the deposition of β-amyloid (Aβ) in senile plaques, the presence of tau protein neurofibrillary tangles and neurodegeneration [2].

Cerebral microbleeds (CMBs) are lesions appearing as hyposignals on susceptibility weighted imaging (SWI) magnetic resonance sequences that reflect leakage of blood products and haemosiderin deposits from cerebral vessels damaged by Aβ deposition [3]. They may be related to hypertension or cerebral amyloid angiopathy (CAA). CMBs caused by hypertension have lipohyalinosis as a pathological substrate and is predominantly of deep location based on neuroimaging, although CMBs with lobar locations and hypertensive aetiology are frequent. In contrast, CMBs related to amyloid angiopathy are almost exclusively of lobar location [4].

CMBs were initially recognized as an important risk factor for intracerebral haemorrhage (ICH). Progress has been made in the field, and they have been progressively recognized to be related not only to haemorrhagic but also to ischaemic events and, later, as an important factor contributing to cognitive decline [5, 6]. In patients with AD, CMBs were initially considered clinically silent, but their clinical role is becoming increasingly recognized. CMBs are relatively frequent in AD and are mainly related to CAA [7, 8]. AD patients with CMBs had lower CSF Aβ42 and Aβ40 levels than AD patients without CMBs, especially in those patients with a CMB pattern suggestive of CAA [9, 10]. Similar results were observed in nonamnestic mild cognitive impairment (MCI) patients [11].

In an extensive cohort of persons without dementia, the presence of numerous CMBs, especially in strictly lobar locations, was associated with worse performance on tests measuring cognitive function [12]. However, contradictory data have been published about CMBs and changes in cognition in AD patients [13–16].

Multiple CMBs predicted mortality in patients with AD [17]. In populations enriched for vascular disease, lobar CMBs have been found to increase the risk for stroke-related mortality [18] and, more specifically, mortality due to ICH, indicating that these patients should be treated with the utmost care [19]. Subsequently, the presence of nonlobar CMBs was associated with an increased risk for cardiovascular events and cardiovascular mortality [20].

Considering the relationship of CMBs and AD, the aims of this study were i) to investigate the influence of CMBs on the incidence of stroke and mortality, ii) to evaluate the impact of CMBs on the cognitive evolution of patients with mild-moderate AD, iii) to evaluate whether the number and localization of CMBs was associated with the cognitive decline of AD patients, and iv) to analyse the relationship of APOE genotype and CSF AD biomarkers to the presence and localization of CMBs.

## Material and methods

### Patients

Patients were consecutively and prospectively recruited from the Cognitive Disorders Unit of the Hospital Universitari Santa Maria (Lleida, Spain) from November 2014 to November 2017 in accordance with the protocol of NCT02814045. Patients with evaluable MRI results were included in the present study.

Eligible patients 1) were males and females above 60 years without specific treatment for dementia at the moment of the inclusion, with a diagnosis of mild or moderate AD (Mini-Mental State Examination ≥20) according to the National Institute on Aging-Alzheimer’s Association (NIA-AA) criteria [21]; 2) had an absence of visual and hearing problems that, in the investigator’s judgement, would have made it difficult to comply with the study protocols; 3) provided an informed consent form signed by the patient and the responsible caregiver; and 4) had a knowledgeable and reliable caregiver who accompanied the patient to all clinic visits during the study.

The exclusion criteria included the following: 1) previous diagnosis of OSA; 2) severe AD, other types of dementia or mild-moderate AD with current acetylcholinesterase inhibitor or memantine treatment. All exclusion criteria are available in the protocol for NCT02814045.

At baseline, patients underwent brain magnetic resonance imaging (MRI), and blood and CSF samples were obtained to determine APOE genotypes and levels of Aβ42, total tau (t-tau) and p-tau, respectively. A complete neuropsychological battery was performed at baseline and after 1 year of follow-up. The patients who agreed were followed for 3 years according to usual clinical practice. In these patients, the MMSE was used as a tool for cognitive monitoring at 24 months.

After completing the baseline visit and all complementary exams, the patients received acetylcholinesterase inhibitor treatment at the discretion of the physician.

### Sociodemographic and clinical variables

Clinical history and sociodemographic data of the patients was recorded. The patients were followed up for a maximum of 80 months to assess the risk of cerebrovascular events and death from any cause. The incidence of stroke, including ischaemic, haemorrhagic or TIA, was recorded, and these types of stroke were all classified together as a stroke. We also recorded information on antithrombotic medication (i.e., antiplatelet and/or anticoagulant). In addition, we obtained information on mortality from the medical records (deceased: yes or no). We examined the cause of death and classified it as cardiovascular mortality or not cardiovascular. Regarding cardiovascular mortality, we included stroke, myocardial infarction and other venous or arterial thromboembolism events.

### Neuropsychological battery

The patients underwent a neuropsychological evaluation at the beginning of the study and after one and 2 years of follow-up. The following assessments were obtained at baseline and at 12 months: Mini-Mental State Examination (MMSE), Hachinski scale, Wechsler Adult Intelligence Scale (WAIS-III) digit span test; Stroop colour word interference test (Stroop); verbal fluency test; Trail Making Test (TMT) A and B; California verbal learning test (CVLT), Rey-Osterrieth complex figure test (RCFT), Cornell depression scale, neuropsychiatric inventory, Zarit scale and EuroQol test. The MMSE was the primary endpoint, and it was also performed at 24 months [22, 23].

### Brain MRI

All patients were evaluated by MRI performed using a Siemens Avanto 1.5 T MRI scanner. The protocol performed included volumetric T1-weighted 3D MPR (FoV: 256 mm, slice thickness: 1 mm, TR: 1600 ms, TE: 3.01 ms, and distance factor: 50%); T2 TSE axial (FoV: 230 mm, slice thickness: 5 mm, TR: 3560 ms, TE: 89 ms, and distance factor: 30%); FLAIR axial (FoV: 230 mm, slice thickness: 5 mm, TR: 8000 ms, TE: 94 ms, and distance factor: 30%); T2 gradient echo axial (FoV: 230 mm, slice thickness: 5 mm, TR: 800 ms, TE: 26 ms, and distance factor: 30%); echo planar diffusion images (FoV: 230 mm, slice thickness: 5 mm, TR: 3902 ms, TE: 102 ms, and distance factor: 40%); T2 TSE coronal (FoV: 210 mm, slice thickness: 5 mm, TR: 3250 ms, TE: 100 ms, and distance factor: 20%).

The presence and number of CMBs was evaluated manually by two independent reviewers on axial SWI images according to current consensus criteria [24] and categorized as lobar (i.e., cortical-subcortical), deep (i.e., basal ganglia, thalamus or brainstem), or mixed (any lobar). The total number of CMBs was also recorded.

### Genetic analysis

Blood samples were obtained to determine APOE genotypes. DNA was extracted from the buffy coat cells using a Maxwell® RCS Blood DNA Kit (Promega, USA), and 20 μL of DNA was used for genotyping by polymerase chain reaction (PCR).

### CSF biomarker analysis

Patients underwent lumbar puncture between 8:00 and 10:00 a.m. to avoid variations related to the circadian rhythm. Samples were collected in polypropylene tubes, centrifuged at 2000 xg for 10 min at 4 °C and stored at − 80 °C until use. The levels of CSF Aβ42 (Innotest® β-Amyloid (1–42)), t-tau (Innotest® hTAU Ag) and p-tau (Innotest® Phospho-Tau (181P)) were determined by the enzyme immunoassay method in accordance with the manufacturer’s instructions. All samples were measured in duplicate, and the values were expressed in pg/ml.

### Statistical analysis

Descriptive statistics consisting of the mean (standard deviation) or median (interquartile range) were estimated for quantitative variables with a normal or non-normal distribution, respectively. The absolute and relative frequencies were used for qualitative variables. The normality of the distribution was assessed using the Shapiro–Wilk test.

A bivariate analysis was performed for the demographic and clinical data of the patients with CMBs compared to the patients without CMBs using Student’s t test or a nonparametric Mann–Whitney U test for quantitative variables (depending on the distribution of the data) and Fisher’s exact test for qualitative variables. The short-term change in cognition measurements (after 12 months of follow-up) was compared between groups (CMB or non-CMB) using linear models that included a group term and an adjustment for the baseline variables that could potentially affect both cognition and the presence of microbleeding independently that did not show significant differences in the bivariate study (age, sex, BMI, hypertension, cardiopathy, education level and antialzheimer pharmacological therapy). The change in MMSE scores over the long term (after 2 years of follow-up) was compared between groups using a linear mixed-effects model.

The risk of death and stroke during the follow-up was evaluated by a log rank test.

Data management and statistical analyses were performed by the SPSS 19.0 program (SPSS, Chicago, IL).

## Results

**Fig. 1:**
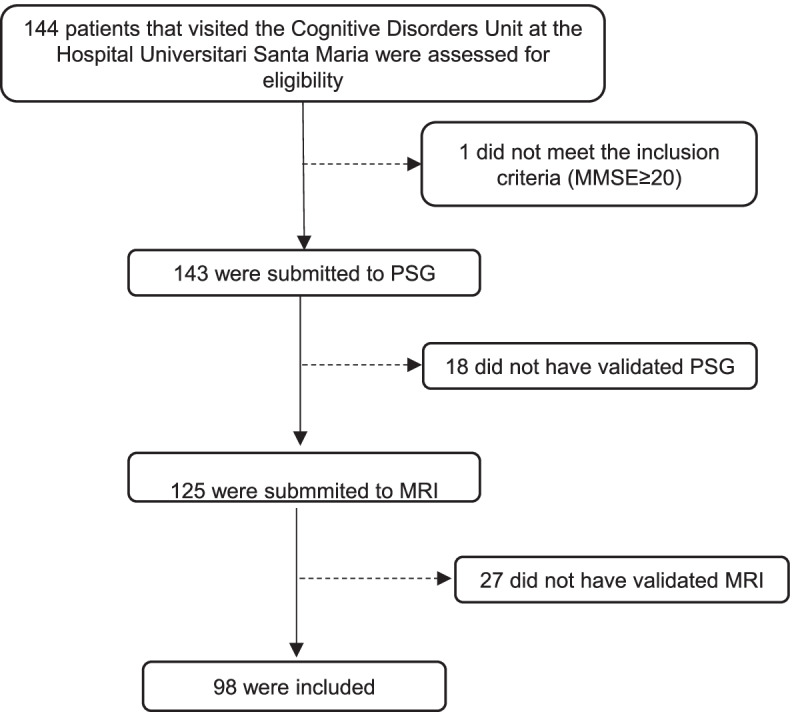
Flow diagram showing the enrollment of subjects

The cohort included 98 consecutive patients with mild-moderate AD (Fig. 1), most of whom were women (58.2%), with a mean age of 75.4 (5.45) years and a BMI of 27.6 (3.64). Hypertension was the most frequent vascular risk factor (54.1%), followed by dyslipidaemia (42.1%) and diabetes mellitus (24.5%). Forty-one patients (41.8%) were taken statins, 19 patients (19.4%) were on oral hypoglycemic agents and 47 (47.9%) were on treatment with antihypertensive medications. Forty-five of the subjects were APOE **ε**4 positive. Table 1 summarizes all of the demographic characteristics, including basal cognitive status, according to the presence of CMBs.

**Table 1:** Characteristics of Alzheimer’s disease patients according the presence of microbleeds

	Number of patients (% of patients)			
	Total cohort (*n* = 98)	No Microbleeds (*n* = 50)	Microbleeds (*n* = 48)	*p* value No microbleeds vs microbleeds
Female	57 (58.2)	34 (68.0)	23 (47.9)	0.044
Age, mean (SD), y	75.4 (5.45)	74.4 (5.69)	76.4 (5.06)	0.475
MMSE score, mean (SD)	23. 90 (3.45)	24.17 (3.66)	23.87 (2.92)	0.074
Hypertension	53 (54.1)	26 (52.0)	27 (56.3)	0.673
Hypercholesterolemia	43 (42.14)	25 (50.0)	17 (35.4)	0.145
Diabetes mellitus	24 (24.5)	11 (22.0%)	13 (27.1)	0.559
Current smoking	3 (2.94)	0 (0)	3 (6.3)	0.175
*Blood pressure, mean (SD), mm/Hg*				
Systolic	140.06 (19.13)	139.69 (19.55)	140.32 (18.71)	0.439
Diastolic	82.55 (10.13)	82.04 (9.89)	82.71 (10.50)	0.764
BMI, mean (SD)	27.56 (3.64)	27.66 (3.75)	27.51 (3.62)	0.858
Lacune presence				
WMH presence (Fazekas ≥2)	32 (31.36)	11 (22.0)	21 (43.8)	0.022
Antiplatelet drugs	22 (21.56)	9 (18.8)	11 (23.4)	0.578
*CSF, mean (SD), pg/mL*				
Aβ42	581.43 (257.82)	594.26 (243.9)	526.88 (209.62)	0.673
Total tau	510.35 (268.95)	500.34 (246.52)	527.02 (295.61)	0.317
pTau	83.30 (52.50)	84.07 (64.41)	83.33 (38.23)	0.736
ApoE ε4	44 (45.8)	24 (51.0)	20 (48.8)	0.314

^*^BMI Body mass index, *CSF* Cerebrospinal fluid, *APOE Ɛ4* Apolipoprotein epsilon 4

A total of 48 (48.9%) patients had at least one CMB. Eight patients (8.2%) had strictly nonlobar CMBs, whereas 39 (40.2%) had any lobar CMB location. Four patients (4.08%) had superficial siderosis. Vascular risk factors (hypertension, diabetes or dyslipidaemia) were not different between patients with and without CMBs. No differences were observed between the patients who took antiplatelet drugs according to the presence of a CMB (55.0% vs. 48.0%; *p* = 0.310) or number of CMBs (2.25 (4.62) vs. 2.32 (5.51); *p* = 0.827).

The patients who presented CMBs had a higher percentage of white matter lesions based on a score equal to or greater than 2 on the Fazekas scale (22.0% vs. 43.8%; *p* = 0.022). No patients with carotid or intracranial stenosis were found.

### Mortality

**Fig. 2:**
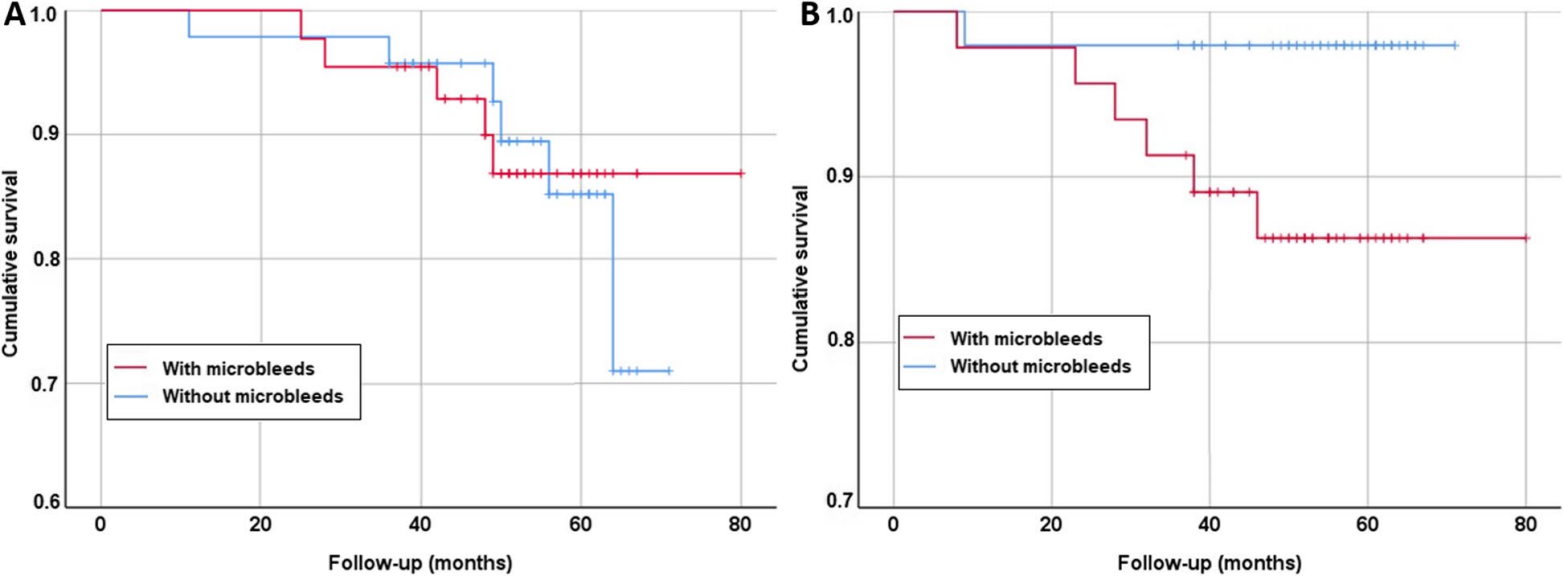
Survival curves based on the presence of microbleeds. **a** Survival curves for global survival; **b** survival curves for incident stroke

The mean follow-up period was 51.5 months, and 11 patients (11.2%) died during follow-up, with only one due to cardiovascular causes and none related to cerebrovascular causes. Mortality did not differ between groups (5 CMB vs. 6 non-CMB; *p* > 0.05) (Fig. 2A).

### Stroke incidence

During the follow-up period, 7 patients had stroke. The stroke incidence was 6.1%. The strokes had the following characteristics: two patients had ischaemic stroke of atherothrombotic aetiology (one of intracranial stenosis and one of vertebrobasilar atheromatosis). Two patients had lacunar ischaemic stroke, and one patient had ischaemic stroke of an undetermined embolic source. One patient had a negative DWI TIA, and there was only one patient with intracerebral haemorrhage. The number of CMBs in patients with stroke was variable but was less than 10 CMBs in all cases (Fig. 1B).

Of the 7 cases of stroke, 6 occurred in patients who had at least one CMB on MRI (log rank < 0.05). All of these CMBs were lobar.

The consumption of antithrombotic drugs did not affect mortality or the incidence of stroke after the follow-up period (*p* > 0.05).

### Biomarkers

We observed a correlation between the total number of CMBs and CSF Aβ42 levels (r = − 0.331; *p* = 0.005). We did not observe differences in the levels of core AD CSF biomarkers in patients with CMBs compared to patients without CMBs or depending on their location for amyloid (lobar: 507.72 pg/mL (209.55) vs. nonlobar: 609.89 pg/mL (200.09); *p* = 0.447), total tau (lobar: 526.54 pg/mL (294.07) vs. nonlobar: 529.11 pg/mL (320.32); *p* = 0.616) or tau pathology (lobar: 85.66 pg/mL (39.53) vs. nonlobar: 73.24 pg/mL (31.98); *p* = 0.585). According to ATN classification, A+ patients had more CMBs than A- patients (1.25 (3.40) vs. 2.97 (6.09), respectively; *p* = 0.031). Similarly, A + T + N+ patients tended to have more CMBs than A-T-N- patients (2.74 (5.81) vs. 0.87 (1.35), respectively; *p* = 0.072). However, we found no differences in location, either by A+ (87.9% lobar in A+ vs. 66.7% lobar in A-; *p* = 0.081) or by A + T + N+ (85.7% lobar in A + T + N+ vs. 71.4% lobar in A-T-N-; *p* = 0.393).

According to APOE **ε**4 status, the age of the patients who were non-APOE **ε**4 carriers was significantly higher (76.7 (4.59) vs. 73.7 (5.82), respectively; *p* = 0.038) (Table 1). In the multivariable analysis, the number of CMBs was associated only with the presence of APOE4. The median number of CMBs was significantly higher in APOE. **ε**4 carriers vs. noncarriers (1.12 (2.87) vs. 3.84 (7.02), respectively; *p* = 0.004). We found no differences in the location of CMBs based on APOE **ε** 4 status (*p* = 0.384).

### Cognition

The mean changes in MMSE scores at the 12-month follow-up was 0.07 (3.04) in the non-CMB group and − 1.50 (2.87) in the CMB group. The difference in the changes between the CMB and non-CMB groups was 1.57 (95% CI: − 0.04 to 3.17; *p* = 0.399) (Table 2). Regarding CMB localization, patients with lobar CMB (23.05(2.24) at baseline and 21.71 (3.10) at 12 month follow-up) haad a greater cognitive impairment than those without CMB (23.00 (2.50) at baseline and 23.03 (3.20) at 12 month follow-up). Similarly, patients with deep CMB (24.00 (3.00) at baseline and 21.33 (2.89) at 12 month follow-up) had a greater cognitive impairment than those without CMB (23.00 (2.50) at baseline and 23.03 (3.20) at 12 mmonth follow-up). The differences in the changes at 12 months for lobar CMB was 1.33 (95% CI − 0.03 to 2.07; *p* = 0.056); 2.67 for deep CMB (95% CI − 1.12 to 6.46; *p* = 0.094); and − 0.031 for the non-CMB group (95% CI − 1.11 to 1.05; *p* = 0.953).

**Table 2:** Comparison between cognitive performance on the cognitive domains evaluated at baseline and after 12 months of follow-up according the presence of CMBs

	No microbleeds	Microbleeds	Difference		
				*p value*	*p value* *adjusted*^*¥*^
	*Mean (sd)*	*Mean (sd)*	*Mean (95% CI)*		
Global cognition					
MMSE	(*n* = 38)	(*n* = 45)			
*Baseline*	23.06 (2.52)	23.17 (2.29)			
*12 months*	23.13 (3.20)	21.67 (3.02)			
*Change*	−0.65 (−1.18 to 1.05)	1.50 (0.58 to 2.71)	1.57 (−0.04 to 3.17)	0.057	0.399
Verbal memory, recognition					
Recognition (CVLT)	(*n* = 39)	(n = 45)			
Baseline	−0.38 (1.43)	−0.80 (1.67)			
12 months	−0.28 (1.34)	−0.56 (2.22)			
Change	0.10 (1.25)	0.24 (2.70)	−0,14 (−1.07 to 0.79)	0.463	0.340
Long-term memory					
Long-term verbal memory, with clues (CVLT)	(*n* = 41)	(*n* = 44)			
Baseline	−1.95 (1.02)	−2.18 (0.89)			
12 months	−1.90 (1.04)	−1.78 (0.90)			
Change	0.05 (1.18)	0.40 (1.19)	−0.35 (−0,86 to 0.16)	0.175	0.403
Long-term visual memory RCFT	(n = 38)	(n = 44)			
Baseline	4.00 (2.60)	4.07 (2.57)			
12 months	4.84 (2.83)	3.64 (2.59)			
Change	0.84 (2.19)	−0.43 (2.45)	1.27 (0.25 to 2.31)	0.016	0.471
Short-term memory					
Short-term verbal memory (CVLT)	(n = 41)	(n = 45)			
Baseline	−1.66 (1.04)	−1.98 (0.75)			
12 months	−2.00 (1.69)	−1.84 (0.74)			
Change	−0.34 (1.79)	0.13 (0.82)	−0.47 (−1.06 to 0.12)	0.113	0.07
Short-term visual memory RCFT	(n = 39)	(n = 44)			
Baseline	4.95 (2.53)	4.64 (2.50)			
12 months	5.23 (2.86)	4.48 (2.77)			
Change	0.28 (2.67)	−0.16 (2.63)	0.44 (− 0.71 to 1.60)	0.451	0.918
CONSTRUCTIONAL PRAXIS					
Copy of the RCFT	(*n* = 40)	(n = 44)			
Baseline	7.35 (3.71)	6.55 (3.87)			
12 months	6.80 (3.88)	6.45 (3.89)			
Change	−0.55 (3.96)	−0.09 (4.07)	− 0,46 (−2.20 to 1.29)	0.602	0.404
EXECUTIVE FUNCTIONS					
Stroop word-color	(n = 40)	(*n* = 43)			
Baseline	7.75 (2.97)	8.23 (3.78)			
12 months	8.43 (3.45)	7.63 (4.36)			
Change	0.68 (3.85)	−0.61 (4.50)	1.29 (− 0.55 to 3.11)	0.170	0.855
Stroop interference	(n = 40)	(*n* = 42)			
Baseline	38.23 (10.06)	41.43 (11.48)			
12 months	42.10 (11.61)	41.60 (14.71)			
Change	3.88 (14.13)	0.17 (17.41)	3.71 (−3.24 to 10.66)	0.294	0.784
Digit span (backwards)	(n = 41)	(n = 45)			
Baseline	8.83 (3.04)	8.78 (2.91)			
12 months	8.44 (3.00)	8.93 (2.44)			
Change	−0.39 (3.37)	0.16 (3.10)	−0.55 (−1.94 to 0.85)	0.822	0.862
TMT-B	(*n* = 11)	(*n* = 6)			
Baseline	6.09 (1.70)	8.50 (2.35)			
12 months	9.00 (3.82)	8.33 (3.33)			
Change	2.91 (4.21)	−0.17 (2.23)	3.08 (−0.25 to 6.40)	0.024	0.832
SPEED PROCESSING					
Stroop words	(n = 40)	(n = 43)			
Baseline	9.43 (3.08)	8.63 (3.36)			
12 months	8.40 (3.28)	7.67 (3.26)			
Change	−1.03 (3.33)	−0.95 (3.48)	− 0,08 (− 1,56 to 1,41)	0.924	0.975
Stroop color	(n = 40)	(n = 43)			
Baseline	10.13 (4.38)	8.98 (4.36)			
12 months	8.25 (3.90)	7.70 (4.27)			
Change	−1.88 (3.52)	−1.28 (4.61)	−0.60 (−2.38 to 1,19)	0.508	0.751
TMT-A	(n = 38)	(n = 42)			
Baseline	6.61 (3.36)	6.45 (3.11)			
12 months	7.21 (3.09)	6.00 (3.39)			
Change	0.61 (3.23)	−0.45 (2.64)	1.06 (−0.27 to 2.38)	0.115	0.107
Behavior					
NPI	(n = 40)	(n = 43)			
Baseline	13.28 (13.19)	9.40 (12.10)			
12 months	13.05 (12.04)	11.00 (13.69)			
Change	−0.23 (14.50)	1.61 (13.37)	−1,84 (−7,94 to 2,28)	0.553	0.642
Cornell	(n = 41)	(n = 42)			
Baseline	11.2 (7.53)	9.31 (6.04)			
12 months	7.85 (6.88)	6.48 (6.66)			
Change	−3.34 (5.36)	−2.83 (6.98)	−0.51 (−3.22 to 2.20)	0.711	0.522
Zarit	(n = 41)	(*n* = 41)			
Baseline	33.02 (17.74)	26.49 (13.01)			
12 months	36.05 (13.68)	38.98 (14.90)			
Change	3.02 (16.38)	12.49 (16.84)	−9.47 (−16.77 to −2.17)	0.012	0.031

Adjusted by baseline, age, sex, BMI, hypertension, cardiopathy, educational level, and pharmacological treatment. BMI, body mass index; CI, confidence interval; MMSE, Mini-mental state examination; ADAS-cog, Alzheimer’s Disease Assessment Scale-Cognitive

^**^MMSE Minimental test, *CVLT* California Verbal Learning Test, *RCF* Rey-Osterrieth Complex Figure Test, *TMT-A* Trail Making Test A, *TMT-B* Trail Making Test B, *NPI* Neuropsychiatric Inventory

An extensive battery of neuropsychological tests was performed to evaluate the different cognitive domains at the 12-month follow-up. We did not observe significant differences in cognitive subdomains, such memory, executive functioning, speed processing, or behaviour (Table 2).

According to MMSE scores at the 2-year follow-up, significant cognitive impairments were observed in both groups (*p* < 0.001). However, the changes between 12 and 24 months of follow-up were similar between the CMB and non-CMB groups (*p* = 0.202). So, after 24 months of follow-up, the patients with CMBs did not worsened more than non CMBs group (*p* = 0.564).

## Care burden

We found a greater increase in the Zarit scale scores of caregivers of the CMB patients (*p* = 0.031) after 12-month follow-up (Table 2).

## Discussion

In this study, we carried out a multimodal approach to evaluate the impact of CMBs at clinical, neuropsychological, and survival levels, as well as on AD biomarkers in the CSF in patients with mild-moderate AD. We observed a high prevalence of CMBs in our study population. The patients with CMBs presented a higher risk of stroke without showing a higher mortality rate after 80 months of follow-up. At the cognitive level, we did not observe differences depending on the presence or absence of CMBs, except an increase in caregiver burden possibly associated with increased risk of stroke in the CMB group after 1 year of follow-up. CMBs patients deteriorated more rapidly at 12 months according to MMSE scores, with no differences observed at 24 months. At the level of biomarkers, the presence of cerebral amyloidosis and APOE ε4 were associated with a greater presence of CMBs, and this association was not observed with markers of tauopathy or neuronal degeneration.

In our study, we found a prevalence of CMBs of 48.9%. This prevalence was slightly higher than that observed in other studies, which ranged between 24 and 37.3% [25, 26]. In a recent study with MCI patients, the prevalence of CMBs was 45% and reached 67% in subjects with an amnesic profile [11]. In patients with other types of dementia, such as primary progressive aphasia, a prevalence of 28% was observed and reached 50% in cases of logopenic aphasia [3, 27] or between 16.9 and 45.2% in Lewy body dementia [26, 28–30].

A previous study found that CMBs with lobar localizations increased the risk for incident stroke and stroke-related mortality, whereas nonlobar CMBs were associated with an increased risk for cardiovascular events and cardiovascular mortality [17, 20]. In our study, only 1 death of cardiovascular cause was observed after 80 months of follow-up; therefore, despite the long follow-up period, we cannot draw conclusions about the risk of death in AD patients with CMBs. We observed 6 cases of stroke in the CMB group, showing an increased risk of stroke in this population. All of these patients had lobar CMBs. We did not find that the use of antithrombotic drugs increased the risk of lobar and nonlobar CMBs, contrary to what the Mistral study observed [20].

It was tempting to assume that the stroke incidence in patients with AD might be related to lobar ICH, but in our study, there was only one patient who had lobar ICH, in contrast to six cases of ischaemic stroke. All the patients who suffered stroke had fewer than 10 CMBs, and these were in all cases with lobar locations. Among the patients with more than 10 CMBs, no patient suffered a stroke. Taking into account these data and other studies, it might be hypothesized that stroke in the AD patients in our study was more related to cardiovascular risk factors or white matter lesions rather than the presence of CMBs due to Aβ load itself and that the incidence of ICH in patients with AD is related not only to CMBs prevalence but also to concomitant use of antithrombotic medications [20].

Theoretically, small vessel disease, and concretely CMBs, could contribute to cognitive decline in AD and not only in patients with vascular dementia. However, this relationship remains controversial. In a previous study, Li et al., in a cohort of AD patients over 8 years of follow-up, observed differences in cognitive performance between groups with or without CMBs. The decline was noted both in global cognitive function and in executive function and memory, but the differences were observed only in the group with CMBs with lobar locations, especially those located in the temporal lobe [13]. However, there are other studies that have not shown any relationship between the presence of CMBs and cognition [14]. In particular, Van der Vlies et al. [15] found that there was no relationship between CMB presence and rates of decline in MMSE scores in a follow-up of 3 years. In a previous meta-analysis [16], no differences in neuropsychological assessment were found in patients with AD with and without CMBs. Similarly, we found no differences in cognitive decline for global cognition or specific cognitive domains. We observed only that patients with CMBs performed worse on the MMSE after 12 months of follow-up, but these differences were not found at 24 months, suggesting that the presence of CMBs may initially accelerate cognitive decline, but we believe that these results should be taken with caution. We did observe that the caregivers of patients with CMBs presented greater overload according to the Zarit scale than the caregivers of patients with AD without CMBs. Since there were no differences in cognition, this increase in workload could be explained by the increase in stroke. Benedictus et al. showed that CMBs in AD patients were associated with an increased risk for institutionalization. This may be explained by the strong predictive value of (lobar) CMBs for future stroke-related mortality [20]. In line with previous findings, our current results indicated that patients with AD with CMBs were particularly vulnerable to future events rather than to more rapid gradual cognitive or functional decline [15].

Finally, since lobar CMBs are related to CAA caused by Aβ40 deposits, we evaluated the results of the different AD core biomarkers in CSF and the relationship with APOE. Similar to other studies, we found that the presence of Aβ42 was associated with a higher risk of CMBs. We also observed that those patients with the 3 positive biomarkers (amyloid, total tau and p-tau) presented a higher number of CMBs. These results have been observed in other studies, both in the population with AD [8, 9, 31] and in patients with MCI [10].

APOE **ε** 4 is the main genetic predisposition marker for late-onset AD. We observed that the number of CMBs was associated with the presence of ApOE **ε** 4 and that the median number of CMBs was significantly higher in ApOE carriers than in noncarriers regardless of location. Several studies have shown that APOE **ε**4 AD patients have a higher risk of CMBs and white matter lesions, showing that APOE **ε** 4 reduces the integrity of amyloid-damaged cerebral vasculature, increasing the risk of stroke and cerebral haemorrhage [32, 33].

This study has several limitations. First, the low cognitive impairment observed during the first year of follow-up may have also limited the finding of significant differences. Since the study was designed for 1 year of follow-up, we had data for global cognition supported by the MMSE, but we did not have data on the different cognitive subdomains after 2 years of follow-up. We used an inclusion threshold of MMSE> 20; therefore, the results should be extrapolated cautiously to those with more advanced stages of the disease. The patients in this study were enrolled from a cognitive unit and not from a population-based community. Regarding the study of CSF biomarkers, Aβ40 levels (related to cerebral amyloid angiopathy) were not quantified. Finally, we recruited a relatively small sample size, which could have missed some associations, such as a hypothetical relationship between mortality and CMBs.

## Conclusions

Based on our results, we can conclude that CMBs were frequent in AD patients. CMBs increased the risk of ischaemic stroke in AD patients, but no changes in mortality were observed. The patients with CMBs did not seem to have a different evolution of cognitive decline with the exception of an increase in caregiver overload. Based on these findings and prior knowledge, CMBs seem to be a marker of vulnerability in AD patients rather than a marker for more rapid gradual cognitive or functional decline.

## Data Availability

The datasets used and analysed during the current study available from the corresponding author on reasonable request.
